# The Liability of Voluntary Hospitals for Negligence

**Published:** 1904-01-02

**Authors:** 


### THE LIABILITY OF VOLUNTARY HOSPITALS FOR NEGLIGENCE.

WHEN one recalls the thousands of patients who pass through the wards of our hospitals in the course of a year, not all of whom are cured nor the subjects of successful operations, the wonder of it is that one hears so little of actions for negligence being brought against these institutions. What is the meaning of this curious fact? Is it that our hospitals are so efficient that a patient hesitates before incurring the odium of appearing before a Court of Law, or is it that the authorities are by law divested of all responsibility? If the hospitals are not responsible they can hardly point to this absence of litigation as a tribute to their efficiency, and, on the other hand, they are entitled to lay claim to a high degree of efficiency if in fact they are legally responsible.

We will now proceed to consider the interesting legal question whether voluntary hospitals are liable in damages for injury to a patient caused by the negligence or want of skill of their medical, surgical, or nursing staff. In a previous article we had occasion to deal with this matter, our attention having been called to the subject owing to certain New York hospitals having been sued for the alleged negligence of their staff. On that occasion we based our conclusions, too confidently perhaps, on some decisions in the American Courts which appeared to finally dispose of the question. On looking again into the matter we find that not only do the American decisions conflict with each other, but some of them omit any reference to important English cases which bear upon the question at issue.

From the standpoint of English law there is no doubt whatever that a medical man whether he be remunerated or not, is personally liable for any negligence or want of skill which he may display in treating a patient. In order to ascertain whether the hospital committee of whose staff he is a member is also liable, it is necessary to examine in what exact relation he stands to the governing body of that hospital. Where he is employed for a fixed period and at a definite remuneration, the relationship of master and servant clearly exists, and the maxim *respondeat superior* would apply; in that case the only question to determine is whether the hospital by being a charitable institution, supported by voluntary contributions, can claim exemption from liability on that ground.

Going back to first principles, it is sound law that a breach of a trust undertaken voluntarily will be a good ground for an action. This principle was acted upon by Holt, C. J., in the famous case of *Coggs* v. *Bernard*, in which it was decided that if a man undertakes to carry goods safely and securely, he is responsible for any damage they may sustain in the carriage through his neglect, though he was not a common carrier and was to have nothing for the carriage. This case is known as that of the gratuitous bailee. Applying this case to a voluntary hospital, there appears to be no distinction in principle. The patient, who may be called the bailor, entrusts his person instead of his goods to the care of the institution. So it would seem that although receiving no reward the hospital would be responsible for any injury to the patient through its neglect. It may, however, be urged that although technically responsible, yet the charitable funds would not be applicable to the payment of damages, because otherwise money devoted to a charitable object would be diverted in breach of trust. This is a specious argument, but has found favour in some of the American Courts, which have relied upon some dicta of Lord Cottenham in the Scotch case of *Duncan* v. *Findlater*, which was decided by the House of Lords in 1839. Its unsoundness was, however, exposed by Lord Westbury in his judgment in the same House in the later case of *Mersey Docks Trustees* v. *Gibbs*, and since this decision the point would probably be considered beyond argument in an English Court of law.

We come to the conclusion, therefore, that if the relation of master and servant does in fact exist between the hospital and the negligent doctor or nurse, not only is the doctor or nurse liable, but also the hospital itself. The only other difficulty which remains is as to the position of the honorary medical staff. Does the relation of master and servant exist in that case? As a rule they are elected for some specified period, and are liable to removal in special circumstances during that period, but they would, no doubt, resent any interference by the governing body with them in the performance of their medical duties. It is, we believe, becoming more and more the practice to pay the medical staff a nominal salary in order to give the governing body more control, and it is perhaps hard to say that those who are not paid stand in the relation of servants to the hospital. But after all this does not in our opinion relieve the hospital of liability. If not servants they are surely agents of the institution to carry out its work and if, in the course of such work, they are negligent, the institution upon whose behalf they act, and whom in a sense they represent, as agents, is equally liable as being their principal.

